# Crystal structure of 4-[4-(eth­oxy­carbon­yl)piperazin-1-yl]benzoic acid

**DOI:** 10.1107/S2056989016012482

**Published:** 2016-08-09

**Authors:** Md. Serajul Haque Faizi, Musheer Ahmad, Irina A. Golenya

**Affiliations:** a Department of Chemistry, Indian Institute of Technology Kanpur, Kanpur, UP 208 016, India; b Department of Applied Chemistry, Aligarh Muslim University, Aligarh 202 002, India; c Department of Chemistry, Taras Shevchenko National University of Kyiv, 64 Vladimirska Str., Kiev 01601, Ukraine

**Keywords:** crystal structure, piperazine, hydrogen bonds, carb­oxy­lic acids, 4-fluoro­benzoic acid, ethyl 1-piperazine­carboxyl­ate

## Abstract

The asymmetric unit of the title compound, C_14_H_18_N_2_O_4_, contains two independent mol­ecules (*A* and *B*) which have essentially the same conformation. The piperazine rings adopts chair conformations with the N atoms out of plane. The dihedral angles formed by the four approximately planar C atoms of the piperazine ring and the benzene ring is 30.8 (5)° in mol­ecule *A* and 30.6 (5)° in mol­ecule *B*. In the crystal, mol­ecules *A* and *B* are connected by a pair of O—H⋯O hydrogen bonds, forming a dimer with graph-set notation *R*_2_^2^(8). Weak C—H⋯O hydrogen bonds connect the dimers, forming zigzag chains along [001].

## Abstract 2

4-[4-(Eth­oxy­carbon­yl)piperazin-1-yl]benzoic acid (EPBA) is the product of a reaction between ethyl 1-piperazine­carboxyl­ate and 4-fluoro­benzoic acid. The conformation of the two independent mol­ecules (*A* and *B*) in the asymmetric unit is similar. The piperazine ring adopts a chair conformation in both mol­ecules. The dihedral angles formed by the four approximately planar C atoms of the piperazine ring and the benzene ring is 30.8 (5)° in mol­ecule *A* and 30.6 (5)% in mol­ecule *B*.

## Chemical context   

Piperazines are among the most important building blocks in drug discovery today. The piperazine nucleus is capable of binding to multiple receptors with high affinity and therefore piperazine has been classified as a privileged structure (Dinsmore & Beshore, 2002 ▸). Piperazine and its derivatives are important pharmacores that can be found in biologically active compounds across a number of different therapeutic areas (Berkheij *et al.*, 2005 ▸), such as anti­fungal (Upadhayaya *et al.*, 2004 ▸), anti-bacterial, anti­malarial, anti-psychotic agents (Chaudhary *et al.*, 2006 ▸), HIV protease inhibitors (Dorsey *et al.*, 1994 ▸), anti-depressant and anti-tumour activity against colon, prostate, breast, lung and leukemia tumors (Hulme & Cherrier, 1999 ▸). A review on the current pharmacological and toxicological information for piperazine derivatives is given by Elliott (2011 ▸). The title compound also contains a carb­oxy­lic group, which has been widely used in various fields such as coordination chemistry (Rueff *et al.*, 2001 ▸), pharmaceutical chemistry (Strachan *et al.*, 2007 ▸) and supra­molecular chemistry (Desiraju, 2002 ▸). Recently, the main focus for carb­oxy­lic acids has been in crystal engineering *via* hydrogen-bonded assembly of organic acids and organic bases (Grossel *et al.*, 2006 ▸). In an attempt to further synthesis piperazine derivatives, the title compound was synthesized and the crystal structure is reported herein.

## Structural commentary   

**Figure 1:**
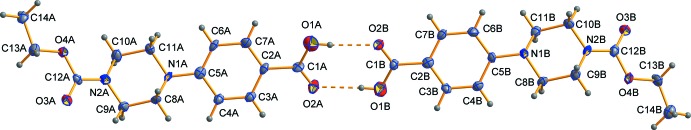
The mol­ecular structures of the two crystallographically independent mol­ecules (*A* and *B*) in the asymmetric unit of the title compound. Displacement ellipsoids are drawn at the 40% probability level. Hydrogen bonds are shown as dashed lines.

The mol­ecular structure of the asymmetric unit is shown in Fig. 1 ▸. The conformation of the two mol­ecules (*A* and *B*) is essentially the same. The piperazine rings are in chair conformations with the N atoms (N1*A*/N2*A* and N1*B*/N2*B*) out of plane of the essentially planar C atoms. The dihedral angles formed by the four approximately planar C atoms of the piperazine ring (C8*A–*C11*A* and C8*B*–C11*B*) and the benzene ring (C2*A*–C7*A* and C2*B*–C7*B*) is 30.8 (5)° in mol­ecule *A* and 30.6 (5)° in mol­ecule *B*.
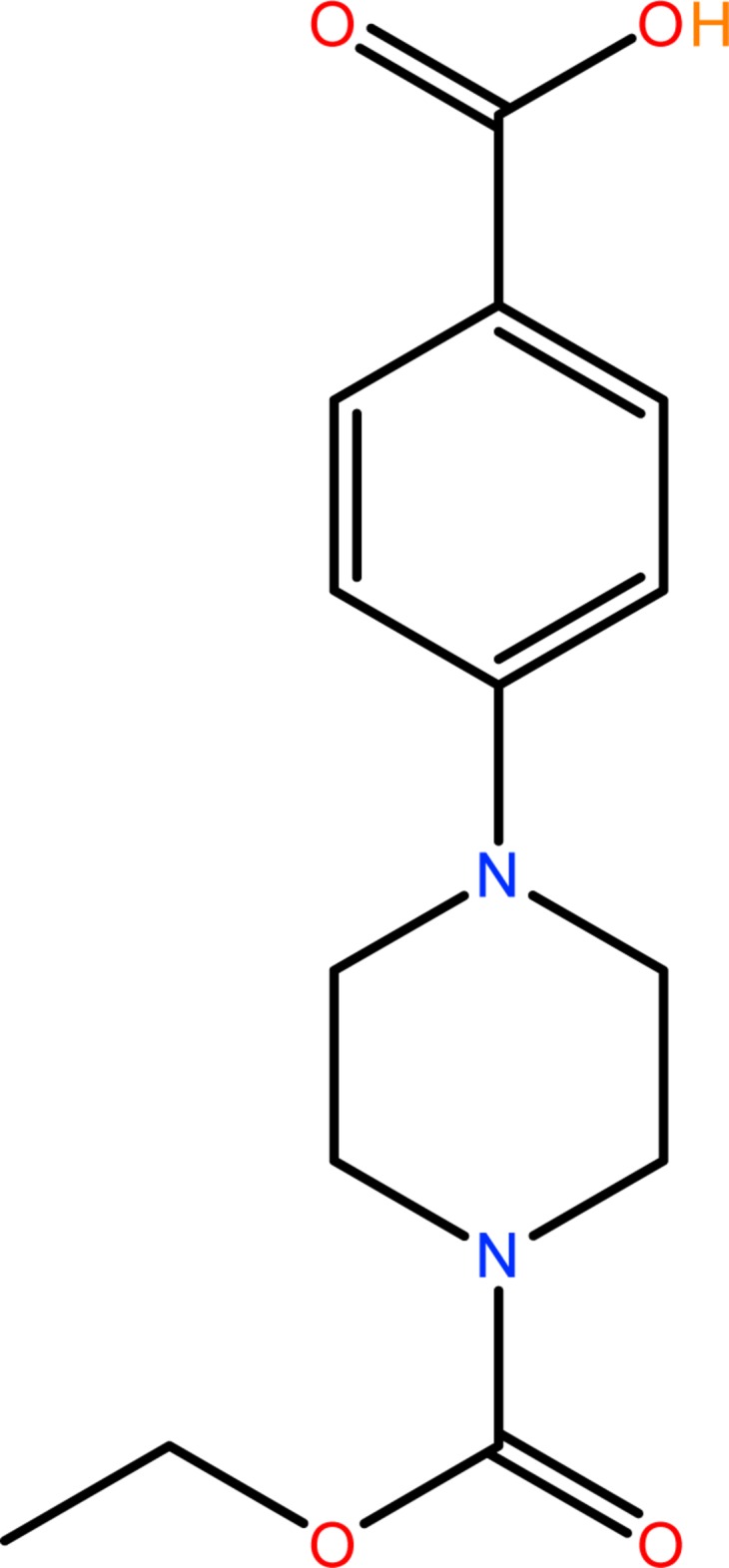


## Supra­molecular features   

**Figure 2:**
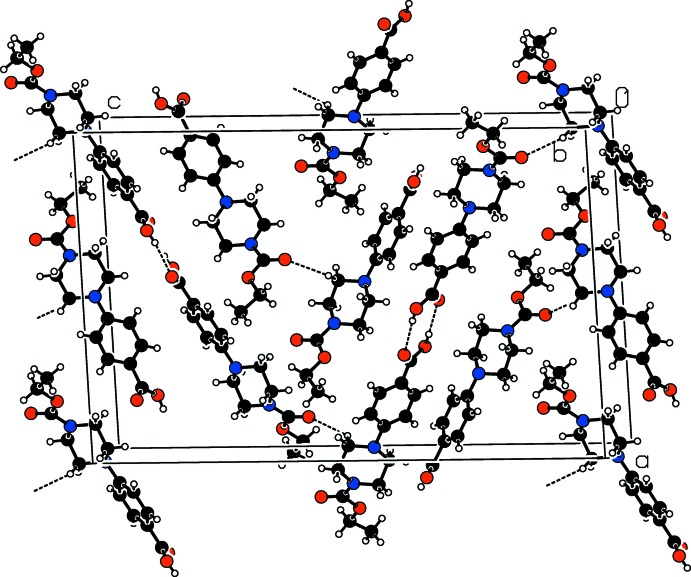
Part of the crystal structure with O—H⋯O and weak C—H⋯O hydrogen bonds shown as dashed lines.

In the crystal, mol­ecules *A* and *B* are connected by a pair of O—H⋯O hydrogen bonds (Fig. 1 ▸, Table 1 ▸), forming a dimer with graph set 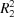
(8). In addition, weak C—H⋯O hydrogen bonds connect the dimers, forming zigzag chains along [001] (Fig. 2 ▸).

## Database survey   

A search of the Cambridge Structural Database (CSD, Version 5.37, update February 2015; Groom *et al.*, 2016 ▸) revealed 11 crystal structures containing the (eth­oxy­carbon­yl)piperazin-1-yl group. Three of these also contain a benzene ring attached to the other piperazine N atom *viz*. ethyl 4-(5-bromo-2-formyl­phen­yl)piperazine-1-carboxyl­ate (EPEPUL; Nour *et al.*, 2011 ▸), ethyl 4-[4-nitro-2-(tri­fluoro­meth­yl)phen­yl]piperazine-1-carboxyl­ate (OMOJAB; Lynch & McClenaghan, 2004 ▸) and ethyl 4-[2-nitro-4-(tri­fluoro­meth­yl)phen­yl]piperazine-1-carboxyl­ate (OMOJEF; Lynch & McClenaghan, 2004 ▸). The dihedral angles formed by the four essentially planar C atoms of the piperazine ring and the benzene ring are 48.4 (1)° for EPEPUL, 44.1 (1)° for OMOJAB and 43.2 (2) and 43.7 (2)° for the two independent mol­ecules in OMOJEF.

## Synthesis and crystallization   

The title compound was prepared by a mixture of ethyl 1-piperazine­carboxyl­ate (2.0 g, 12.6 mmol), 4-fluoro­benzoic acid (1.7 g, 12.6 mmol), and K_2_CO_3_ (2.6 g, 18.9 mmol) in 10 mL of dry aceto­nitrile which was heated at 353 K for 12 h with constant stirring under a nitro­gen atmosphere. After cooling to room temperature, the mixture was poured slowly onto ice-cold water (100 ml) and acidified with glacial acetic acid (AcOH) to pH 3–5. After filtration, the product was obtained as a pale-white crystalline solid (70%). Crystals of the title compound used for X-ray analysis were obtained within three days by slow evaporation of the aceto­nitrile solvent.

## Refinement   

Crystal data, data collection and structure refinement details are summarized in Table 2 ▸. H atoms were placed in calculated positions with C—H = 0.95–0.99 Å, O—H = 0.84 Å and included in the refinement in a riding-motion approximation with U_iso_(H) = 1.2*U*_eq_(C) or 1.5*U*_eq_(O, C_meth­yl_). The crystal quality was generally poor and although the best crystal available was selected, the precision of the structure has been affected by the crystal quality.

## Supplementary Material

Crystal structure: contains datablock(s) I. DOI: 10.1107/S2056989016012482/lh5818sup1.cif

Structure factors: contains datablock(s) I. DOI: 10.1107/S2056989016012482/lh5818Isup2.hkl

Supporting information file. DOI: 10.1107/S2056989016012482/lh5818Isup3.cml

CCDC reference: 1497342

Additional supporting information: crystallographic information; 3D view; checkCIF report

## Figures and Tables

**Table 1:** Hydrogen-bond geometry (Å, °)

*D*—H⋯*A*	*D*—H	H⋯*A*	*D*⋯*A*	*D*—H⋯*A*
O1*A*—H1*A*⋯O2*B*	0.84	2.14	2.973 (8)	170
C8*A*—H8*AB*⋯O3*B* ^i^	0.99	2.56	3.225 (11)	124
O1*B*—H1*B*⋯O2*A*	0.84	2.11	2.934 (8)	168

Symmetry code: (i) 
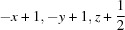
.

**Table 2:** Experimental details

Crystal data	
Chemical formula	C_14_H_18_N_2_O_4_
*M*_r_	278.30
Crystal system, space group	Orthorhombic, *P* *n* *a*2_1_
Temperature (K)	173
*a*, *b*, *c* (Å)	18.508 (5), 4.994 (5), 29.594 (5)
*V* (Å^3^)	2735 (3)
*Z*	8
Radiation type	Mo *K*α
μ (mm^−1^)	0.10
Crystal size (mm)	0.29 × 0.21 × 0.15

Data collection	
Diffractometer	Bruker SMART APEX
Absorption correction	Multi-scan (*SADABS*; Bruker, 2003 ▸)
*T*_min_, *T*_max_	0.972, 0.985
No. of measured, independent and observed [*I* > 2σ(*I*)] reflections	12918, 4042, 3100
*R*_int_	0.068
(sin θ/λ)_max_ (Å^−1^)	0.595

Refinement	
*R*[*F* ^2^ > 2σ(*F* ^2^)], *wR*(*F* ^2^), *S*	0.077, 0.202, 1.08
No. of reflections	4042
No. of parameters	363
No. of restraints	1
H-atom treatment	H-atom parameters constrained
Δρ_max_, Δρ_min_ (e Å^−3^)	0.65, −0.47
Absolute structure parameter	0.2 (10)

Computer programs: *SMART* and *SAINT* (Bruker, 2003 ▸), *SIR97* (Altomare *et al.*, 1999 ▸), *SHELXL2014* (Sheldrick, 2015 ▸), *PLATON* (Spek, 2009 ▸) and *DIAMOND* (Brandenburg, 2006 ▸).

## supplementary crystallographic information

### Crystal data

**Table:**

C_14_H_18_N_2_O_4_	*D*_x_ = 1.352 Mg m^−^^3^
*M**_r_* = 278.30	Mo *K*α radiation, λ = 0.71073 Å
Orthorhombic, *P**n**a*2_1_	Cell parameters from 999 reflections
*a* = 18.508 (5) Å	θ = 2.2–25.5°
*b* = 4.994 (5) Å	µ = 0.10 mm^−^^1^
*c* = 29.594 (5) Å	*T* = 173 K
*V* = 2735 (3) Å^3^	Block, colorless
*Z* = 8	0.29 × 0.21 × 0.15 mm
*F*(000) = 1184	

### Data collection

**Table:**

Bruker SMART APEX diffractometer	3100 reflections with *I* > 2σ(*I*)
Radiation source: fine-focus sealed tube	*R*_int_ = 0.068
/w–scans	θ_max_ = 25.0°, θ_min_ = 2.2°
Absorption correction: multi-scan (SADABS; Bruker, 2003)	*h* = −22→21
*T*_min_ = 0.972, *T*_max_ = 0.985	*k* = −5→5
12918 measured reflections	*l* = −35→25
4042 independent reflections	

### Refinement

**Table:**

Refinement on *F*^2^	Hydrogen site location: inferred from neighbouring sites
Least-squares matrix: full	H-atom parameters constrained
*R*[*F*^2^ > 2σ(*F*^2^)] = 0.077	*w* = 1/[σ^2^(*F*_o_^2^) + (0.1131*P*)^2^ + 0.9875*P*] where *P* = (*F*_o_^2^ + 2*F*_c_^2^)/3
*wR*(*F*^2^) = 0.202	(Δ/σ)_max_ = 0.002
*S* = 1.08	Δρ_max_ = 0.65 e Å^−^^3^
4042 reflections	Δρ_min_ = −0.47 e Å^−^^3^
363 parameters	Absolute structure: Flack *x* determined using 850 quotients [(I+)-(I-)]/[(I+)+(I-)] (Parsons *et al.*, 2013)
1 restraint	Absolute structure parameter: 0.2 (10)

### Special details

**Table:**

Geometry. All esds (except the esd in the dihedral angle between two l.s. planes) are estimated using the full covariance matrix. The cell esds are taken into account individually in the estimation of esds in distances, angles and torsion angles; correlations between esds in cell parameters are only used when they are defined by crystal symmetry. An approximate (isotropic) treatment of cell esds is used for estimating esds involving l.s. planes.

### Fractional atomic coordinates and isotropic or equivalent isotropic displacement parameters (Å^2^)

**Table:**

	*x*	*y*	*z*	*U*_iso_*/*U*_eq_	
O1A	0.6918 (3)	0.2291 (13)	0.3795 (3)	0.044 (2)	
H1A	0.6528	0.2795	0.3678	0.065*	
O2A	0.6971 (2)	0.6589 (12)	0.3981 (2)	0.0289 (13)	
O3A	1.1546 (2)	−0.0268 (10)	0.61300 (19)	0.0282 (13)	
O4A	1.1990 (3)	−0.2288 (9)	0.5501 (2)	0.0209 (14)	
N1A	0.9933 (3)	0.2577 (10)	0.4888 (3)	0.0173 (18)	
N2A	1.1101 (3)	0.0764 (13)	0.5433 (2)	0.0260 (15)	
C1A	0.7243 (4)	0.4362 (14)	0.3986 (3)	0.0196 (16)	
C2A	0.7949 (4)	0.3828 (13)	0.4214 (3)	0.0197 (16)	
C3A	0.8164 (3)	0.5513 (14)	0.4560 (3)	0.0217 (16)	
H3AA	0.7865	0.6969	0.4647	0.026*	
C4A	0.8816 (3)	0.5082 (14)	0.4781 (3)	0.0219 (16)	
H4AA	0.8957	0.6237	0.5021	0.026*	
C5A	0.9277 (4)	0.2923 (16)	0.4653 (3)	0.0215 (19)	
C6A	0.9066 (3)	0.1323 (14)	0.4308 (3)	0.0204 (16)	
H6AA	0.9369	−0.0110	0.4216	0.024*	
C7A	0.8404 (4)	0.1743 (18)	0.4086 (3)	0.0212 (16)	
H7AA	0.8266	0.0591	0.3845	0.025*	
C8A	0.9880 (4)	0.2484 (15)	0.5397 (3)	0.022 (2)	
H8AA	0.9564	0.3955	0.5503	0.026*	
H8AB	0.9658	0.0767	0.5490	0.026*	
C9A	1.0603 (4)	0.2747 (17)	0.5609 (3)	0.027 (2)	
H9AA	1.0797	0.4562	0.5551	0.033*	
H9AB	1.0558	0.2519	0.5940	0.033*	
C10A	1.1159 (4)	0.0718 (17)	0.4939 (3)	0.0285 (19)	
H10A	1.1459	−0.0828	0.4846	0.034*	
H10B	1.1402	0.2372	0.4834	0.034*	
C11A	1.0420 (3)	0.0510 (15)	0.4721 (3)	0.0225 (17)	
H11A	1.0470	0.0688	0.4389	0.027*	
H11B	1.0211	−0.1275	0.4785	0.027*	
C12A	1.1547 (3)	−0.0536 (14)	0.5723 (2)	0.0183 (16)	
C13A	1.2448 (4)	−0.3858 (16)	0.5793 (3)	0.0301 (19)	
H13A	1.2154	−0.4868	0.6014	0.036*	
H13B	1.2788	−0.2686	0.5959	0.036*	
C14A	1.2859 (4)	−0.5770 (15)	0.5485 (3)	0.0271 (18)	
H14A	1.3186	−0.6875	0.5667	0.041*	
H14B	1.3141	−0.4739	0.5265	0.041*	
H14C	1.2516	−0.6926	0.5326	0.041*	
O1B	0.5524 (3)	0.7761 (13)	0.3627 (3)	0.045 (2)	
H1B	0.5911	0.7222	0.3744	0.067*	
O2B	0.5460 (2)	0.3407 (11)	0.34180 (19)	0.0242 (12)	
O3B	0.0883 (3)	1.0119 (11)	0.12725 (19)	0.0296 (13)	
O4B	0.0441 (3)	1.2149 (11)	0.1901 (2)	0.0222 (14)	
N1B	0.2507 (3)	0.7383 (12)	0.2493 (3)	0.0208 (19)	
N2B	0.1310 (3)	0.9039 (12)	0.1970 (2)	0.0219 (14)	
C1B	0.5191 (3)	0.5682 (13)	0.3412 (3)	0.0182 (16)	
C2B	0.4492 (4)	0.6208 (14)	0.3179 (3)	0.0194 (16)	
C3B	0.4035 (4)	0.8309 (17)	0.3305 (3)	0.0202 (16)	
H3BA	0.4176	0.9480	0.3543	0.024*	
C4B	0.3379 (4)	0.8706 (15)	0.3089 (3)	0.0205 (16)	
H4BA	0.3066	1.0094	0.3188	0.025*	
C5B	0.3175 (3)	0.7085 (16)	0.2727 (3)	0.0179 (17)	
C6B	0.3630 (4)	0.4965 (14)	0.2608 (3)	0.0237 (16)	
H6BA	0.3492	0.3795	0.2369	0.028*	
C7B	0.4273 (4)	0.4547 (15)	0.2829 (3)	0.0222 (17)	
H7BA	0.4573	0.3093	0.2741	0.027*	
C8B	0.2016 (3)	0.9471 (15)	0.2664 (3)	0.0237 (17)	
H8BA	0.2210	1.1259	0.2586	0.028*	
H8BB	0.1986	0.9348	0.2998	0.028*	
C9B	0.1267 (4)	0.9150 (17)	0.2462 (3)	0.0260 (18)	
H9BA	0.1043	0.7484	0.2578	0.031*	
H9BB	0.0959	1.0677	0.2554	0.031*	
C10B	0.1800 (4)	0.7025 (18)	0.1806 (3)	0.0238 (19)	
H10C	0.1830	0.7130	0.1472	0.029*	
H10D	0.1617	0.5226	0.1888	0.029*	
C11B	0.2552 (4)	0.7435 (14)	0.2011 (3)	0.022 (2)	
H11C	0.2881	0.6001	0.1905	0.026*	
H11D	0.2751	0.9176	0.1910	0.026*	
C12B	0.0880 (4)	1.0370 (15)	0.1678 (3)	0.0241 (18)	
C13B	−0.0015 (4)	1.3737 (16)	0.1605 (3)	0.0266 (18)	
H13C	−0.0346	1.2564	0.1433	0.032*	
H13D	0.0285	1.4762	0.1389	0.032*	
C14B	−0.0436 (4)	1.5605 (15)	0.1900 (3)	0.0276 (18)	
H14E	−0.0708	1.6860	0.1711	0.041*	
H14F	−0.0103	1.6605	0.2095	0.041*	
H14G	−0.0772	1.4574	0.2088	0.041*	

### Atomic displacement parameters (Å^2^)

**Table:**

	*U*^11^	*U*^22^	*U*^33^	*U*^12^	*U*^13^	*U*^23^
O1A	0.047 (4)	0.052 (5)	0.033 (5)	0.000 (3)	0.003 (3)	0.007 (3)
O2A	0.025 (3)	0.024 (3)	0.038 (4)	0.001 (2)	−0.001 (2)	0.006 (3)
O3A	0.025 (3)	0.037 (3)	0.022 (3)	0.003 (2)	−0.005 (2)	−0.003 (3)
O4A	0.020 (3)	0.026 (3)	0.017 (4)	0.002 (2)	0.001 (2)	0.001 (2)
N1A	0.014 (3)	0.010 (4)	0.027 (5)	0.005 (2)	−0.001 (3)	0.000 (2)
N2A	0.025 (3)	0.035 (4)	0.018 (4)	0.004 (3)	−0.001 (3)	−0.003 (3)
C1A	0.024 (4)	0.007 (4)	0.028 (5)	−0.002 (3)	0.002 (3)	0.003 (3)
C2A	0.017 (4)	0.014 (4)	0.028 (5)	0.004 (3)	0.001 (3)	0.004 (3)
C3A	0.018 (3)	0.019 (4)	0.029 (4)	0.000 (3)	0.004 (3)	−0.001 (3)
C4A	0.018 (3)	0.017 (4)	0.030 (4)	0.000 (3)	0.000 (3)	−0.001 (3)
C5A	0.019 (3)	0.018 (4)	0.027 (5)	−0.004 (3)	0.005 (3)	0.006 (4)
C6A	0.016 (3)	0.013 (4)	0.032 (5)	0.005 (3)	0.003 (3)	0.003 (3)
C7A	0.024 (4)	0.026 (4)	0.014 (4)	0.005 (4)	0.003 (3)	0.003 (4)
C8A	0.020 (4)	0.033 (6)	0.012 (5)	0.008 (3)	0.003 (3)	−0.005 (3)
C9A	0.021 (4)	0.034 (5)	0.027 (5)	0.003 (3)	0.001 (3)	−0.009 (4)
C10A	0.022 (4)	0.040 (5)	0.024 (5)	0.005 (3)	0.001 (3)	0.003 (4)
C11A	0.022 (4)	0.025 (4)	0.020 (4)	0.002 (3)	−0.005 (3)	−0.001 (3)
C12A	0.014 (3)	0.024 (4)	0.017 (5)	−0.006 (3)	0.000 (3)	−0.001 (3)
C13A	0.027 (4)	0.029 (5)	0.035 (5)	0.007 (3)	−0.006 (4)	−0.003 (4)
C14A	0.025 (4)	0.016 (4)	0.041 (5)	0.001 (3)	−0.004 (3)	0.000 (3)
O1B	0.032 (3)	0.037 (4)	0.065 (6)	−0.004 (3)	−0.007 (3)	−0.006 (3)
O2B	0.020 (2)	0.020 (3)	0.032 (3)	0.001 (2)	0.002 (2)	−0.001 (3)
O3B	0.029 (3)	0.038 (3)	0.022 (3)	0.007 (2)	−0.003 (2)	−0.005 (3)
O4B	0.020 (3)	0.026 (3)	0.021 (4)	0.008 (2)	−0.003 (2)	0.003 (2)
N1B	0.014 (3)	0.034 (5)	0.015 (4)	0.004 (2)	−0.001 (3)	−0.004 (2)
N2B	0.013 (3)	0.026 (3)	0.026 (4)	0.007 (2)	0.000 (3)	−0.001 (3)
C1B	0.013 (3)	0.018 (4)	0.024 (4)	−0.002 (3)	0.001 (3)	0.000 (3)
C2B	0.023 (4)	0.015 (4)	0.021 (4)	−0.002 (3)	0.004 (3)	0.005 (3)
C3B	0.020 (3)	0.017 (4)	0.024 (4)	−0.004 (3)	−0.004 (3)	−0.002 (3)
C4B	0.021 (3)	0.020 (4)	0.020 (4)	0.002 (3)	0.003 (3)	−0.001 (3)
C5B	0.012 (3)	0.028 (4)	0.014 (4)	0.001 (3)	0.001 (3)	0.000 (3)
C6B	0.025 (4)	0.020 (4)	0.026 (4)	−0.001 (3)	0.000 (3)	−0.007 (3)
C7B	0.019 (3)	0.018 (4)	0.030 (5)	0.005 (3)	0.002 (3)	−0.001 (3)
C8B	0.019 (3)	0.030 (5)	0.022 (4)	0.007 (3)	0.003 (3)	−0.004 (3)
C9B	0.018 (4)	0.035 (5)	0.025 (5)	0.005 (3)	0.000 (3)	−0.001 (3)
C10B	0.023 (4)	0.031 (4)	0.017 (5)	0.003 (3)	−0.001 (3)	−0.002 (4)
C11B	0.019 (4)	0.017 (5)	0.029 (6)	0.001 (3)	0.001 (3)	0.000 (3)
C12B	0.015 (3)	0.023 (4)	0.035 (6)	0.000 (3)	−0.003 (3)	0.000 (3)
C13B	0.030 (4)	0.030 (5)	0.020 (4)	0.006 (3)	−0.006 (3)	0.001 (3)
C14B	0.026 (4)	0.025 (4)	0.032 (5)	0.001 (3)	0.003 (3)	0.008 (3)

### Geometric parameters (Å, º)

**Table:**

O1A—C1A	1.324 (10)	O1B—C1B	1.365 (9)
O1A—H1A	0.8400	O1B—H1B	0.8400
O2A—C1A	1.221 (9)	O2B—C1B	1.241 (9)
O3A—C12A	1.212 (8)	O3B—C12B	1.207 (9)
O4A—C12A	1.367 (8)	O4B—C12B	1.372 (9)
O4A—C13A	1.441 (9)	O4B—C13B	1.451 (9)
N1A—C5A	1.409 (10)	N1B—C5B	1.425 (10)
N1A—C11A	1.457 (9)	N1B—C11B	1.430 (11)
N1A—C8A	1.510 (10)	N1B—C8B	1.473 (9)
N2A—C12A	1.357 (9)	N2B—C12B	1.351 (9)
N2A—C9A	1.450 (10)	N2B—C10B	1.439 (9)
N2A—C10A	1.464 (10)	N2B—C9B	1.460 (10)
C1A—C2A	1.494 (10)	C1B—C2B	1.490 (10)
C2A—C3A	1.384 (10)	C2B—C7B	1.387 (11)
C2A—C7A	1.392 (11)	C2B—C3B	1.399 (11)
C3A—C4A	1.390 (9)	C3B—C4B	1.388 (10)
C3A—H3AA	0.9500	C3B—H3BA	0.9500
C4A—C5A	1.427 (10)	C4B—C5B	1.394 (11)
C4A—H4AA	0.9500	C4B—H4BA	0.9500
C5A—C6A	1.352 (12)	C5B—C6B	1.399 (10)
C6A—C7A	1.406 (10)	C6B—C7B	1.376 (10)
C6A—H6AA	0.9500	C6B—H6BA	0.9500
C7A—H7AA	0.9500	C7B—H7BA	0.9500
C8A—C9A	1.485 (11)	C8B—C9B	1.519 (9)
C8A—H8AA	0.9900	C8B—H8BA	0.9900
C8A—H8AB	0.9900	C8B—H8BB	0.9900
C9A—H9AA	0.9900	C9B—H9BA	0.9900
C9A—H9AB	0.9900	C9B—H9BB	0.9900
C10A—C11A	1.517 (9)	C10B—C11B	1.532 (11)
C10A—H10A	0.9900	C10B—H10C	0.9900
C10A—H10B	0.9900	C10B—H10D	0.9900
C11A—H11A	0.9900	C11B—H11C	0.9900
C11A—H11B	0.9900	C11B—H11D	0.9900
C13A—C14A	1.523 (11)	C13B—C14B	1.496 (11)
C13A—H13A	0.9900	C13B—H13C	0.9900
C13A—H13B	0.9900	C13B—H13D	0.9900
C14A—H14A	0.9800	C14B—H14E	0.9800
C14A—H14B	0.9800	C14B—H14F	0.9800
C14A—H14C	0.9800	C14B—H14G	0.9800
			
C1A—O1A—H1A	109.5	C1B—O1B—H1B	109.5
C12A—O4A—C13A	114.4 (7)	C12B—O4B—C13B	114.2 (7)
C5A—N1A—C11A	116.8 (7)	C5B—N1B—C11B	115.9 (6)
C5A—N1A—C8A	116.1 (6)	C5B—N1B—C8B	116.2 (7)
C11A—N1A—C8A	110.9 (6)	C11B—N1B—C8B	111.5 (6)
C12A—N2A—C9A	119.0 (7)	C12B—N2B—C10B	119.9 (7)
C12A—N2A—C10A	125.4 (6)	C12B—N2B—C9B	126.0 (6)
C9A—N2A—C10A	114.7 (7)	C10B—N2B—C9B	113.5 (6)
O2A—C1A—O1A	121.2 (7)	O2B—C1B—O1B	120.6 (6)
O2A—C1A—C2A	122.0 (7)	O2B—C1B—C2B	121.1 (6)
O1A—C1A—C2A	116.8 (6)	O1B—C1B—C2B	118.3 (6)
C3A—C2A—C7A	118.9 (6)	C7B—C2B—C3B	118.2 (6)
C3A—C2A—C1A	118.4 (6)	C7B—C2B—C1B	119.7 (6)
C7A—C2A—C1A	122.7 (7)	C3B—C2B—C1B	122.2 (7)
C2A—C3A—C4A	120.3 (6)	C4B—C3B—C2B	120.8 (7)
C2A—C3A—H3AA	119.9	C4B—C3B—H3BA	119.6
C4A—C3A—H3AA	119.9	C2B—C3B—H3BA	119.6
C3A—C4A—C5A	120.7 (7)	C3B—C4B—C5B	120.6 (7)
C3A—C4A—H4AA	119.7	C3B—C4B—H4BA	119.7
C5A—C4A—H4AA	119.7	C5B—C4B—H4BA	119.7
C6A—C5A—N1A	123.3 (7)	C4B—C5B—C6B	118.1 (7)
C6A—C5A—C4A	118.3 (7)	C4B—C5B—N1B	123.2 (7)
N1A—C5A—C4A	118.4 (7)	C6B—C5B—N1B	118.5 (7)
C5A—C6A—C7A	121.1 (7)	C7B—C6B—C5B	121.0 (7)
C5A—C6A—H6AA	119.4	C7B—C6B—H6BA	119.5
C7A—C6A—H6AA	119.4	C5B—C6B—H6BA	119.5
C2A—C7A—C6A	120.7 (7)	C6B—C7B—C2B	121.2 (7)
C2A—C7A—H7AA	119.6	C6B—C7B—H7BA	119.4
C6A—C7A—H7AA	119.6	C2B—C7B—H7BA	119.4
C9A—C8A—N1A	111.2 (7)	N1B—C8B—C9B	110.7 (6)
C9A—C8A—H8AA	109.4	N1B—C8B—H8BA	109.5
N1A—C8A—H8AA	109.4	C9B—C8B—H8BA	109.5
C9A—C8A—H8AB	109.4	N1B—C8B—H8BB	109.5
N1A—C8A—H8AB	109.4	C9B—C8B—H8BB	109.5
H8AA—C8A—H8AB	108.0	H8BA—C8B—H8BB	108.1
N2A—C9A—C8A	111.1 (7)	N2B—C9B—C8B	110.2 (6)
N2A—C9A—H9AA	109.4	N2B—C9B—H9BA	109.6
C8A—C9A—H9AA	109.4	C8B—C9B—H9BA	109.6
N2A—C9A—H9AB	109.4	N2B—C9B—H9BB	109.6
C8A—C9A—H9AB	109.4	C8B—C9B—H9BB	109.6
H9AA—C9A—H9AB	108.0	H9BA—C9B—H9BB	108.1
N2A—C10A—C11A	111.1 (6)	N2B—C10B—C11B	110.2 (6)
N2A—C10A—H10A	109.4	N2B—C10B—H10C	109.6
C11A—C10A—H10A	109.4	C11B—C10B—H10C	109.6
N2A—C10A—H10B	109.4	N2B—C10B—H10D	109.6
C11A—C10A—H10B	109.4	C11B—C10B—H10D	109.6
H10A—C10A—H10B	108.0	H10C—C10B—H10D	108.1
N1A—C11A—C10A	111.4 (6)	N1B—C11B—C10B	109.8 (7)
N1A—C11A—H11A	109.4	N1B—C11B—H11C	109.7
C10A—C11A—H11A	109.4	C10B—C11B—H11C	109.7
N1A—C11A—H11B	109.4	N1B—C11B—H11D	109.7
C10A—C11A—H11B	109.4	C10B—C11B—H11D	109.7
H11A—C11A—H11B	108.0	H11C—C11B—H11D	108.2
O3A—C12A—N2A	125.1 (7)	O3B—C12B—N2B	125.6 (7)
O3A—C12A—O4A	123.3 (7)	O3B—C12B—O4B	123.2 (7)
N2A—C12A—O4A	111.5 (6)	N2B—C12B—O4B	111.1 (7)
O4A—C13A—C14A	106.1 (7)	O4B—C13B—C14B	107.0 (6)
O4A—C13A—H13A	110.5	O4B—C13B—H13C	110.3
C14A—C13A—H13A	110.5	C14B—C13B—H13C	110.3
O4A—C13A—H13B	110.5	O4B—C13B—H13D	110.3
C14A—C13A—H13B	110.5	C14B—C13B—H13D	110.3
H13A—C13A—H13B	108.7	H13C—C13B—H13D	108.6
C13A—C14A—H14A	109.5	C13B—C14B—H14E	109.5
C13A—C14A—H14B	109.5	C13B—C14B—H14F	109.5
H14A—C14A—H14B	109.5	H14E—C14B—H14F	109.5
C13A—C14A—H14C	109.5	C13B—C14B—H14G	109.5
H14A—C14A—H14C	109.5	H14E—C14B—H14G	109.5
H14B—C14A—H14C	109.5	H14F—C14B—H14G	109.5
			
O2A—C1A—C2A—C3A	24.7 (11)	O2B—C1B—C2B—C7B	−24.7 (11)
O1A—C1A—C2A—C3A	−154.3 (7)	O1B—C1B—C2B—C7B	156.3 (7)
O2A—C1A—C2A—C7A	−154.0 (8)	O2B—C1B—C2B—C3B	153.9 (7)
O1A—C1A—C2A—C7A	27.0 (11)	O1B—C1B—C2B—C3B	−25.1 (11)
C7A—C2A—C3A—C4A	−1.4 (11)	C7B—C2B—C3B—C4B	0.4 (11)
C1A—C2A—C3A—C4A	179.8 (6)	C1B—C2B—C3B—C4B	−178.3 (7)
C2A—C3A—C4A—C5A	0.7 (11)	C2B—C3B—C4B—C5B	−2.8 (12)
C11A—N1A—C5A—C6A	4.9 (11)	C3B—C4B—C5B—C6B	3.7 (11)
C8A—N1A—C5A—C6A	−129.0 (8)	C3B—C4B—C5B—N1B	179.7 (7)
C11A—N1A—C5A—C4A	−174.5 (7)	C11B—N1B—C5B—C4B	131.7 (8)
C8A—N1A—C5A—C4A	51.5 (9)	C8B—N1B—C5B—C4B	−2.2 (11)
C3A—C4A—C5A—C6A	0.4 (11)	C11B—N1B—C5B—C6B	−52.3 (9)
C3A—C4A—C5A—N1A	179.9 (7)	C8B—N1B—C5B—C6B	173.8 (7)
N1A—C5A—C6A—C7A	179.7 (7)	C4B—C5B—C6B—C7B	−2.3 (11)
C4A—C5A—C6A—C7A	−0.9 (12)	N1B—C5B—C6B—C7B	−178.6 (7)
C3A—C2A—C7A—C6A	1.0 (12)	C5B—C6B—C7B—C2B	0.1 (12)
C1A—C2A—C7A—C6A	179.7 (7)	C3B—C2B—C7B—C6B	0.9 (11)
C5A—C6A—C7A—C2A	0.1 (12)	C1B—C2B—C7B—C6B	179.6 (7)
C5A—N1A—C8A—C9A	−166.9 (6)	C5B—N1B—C8B—C9B	−166.6 (7)
C11A—N1A—C8A—C9A	56.5 (8)	C11B—N1B—C8B—C9B	57.6 (8)
C12A—N2A—C9A—C8A	−137.3 (8)	C12B—N2B—C9B—C8B	−134.8 (7)
C10A—N2A—C9A—C8A	53.3 (9)	C10B—N2B—C9B—C8B	54.0 (8)
N1A—C8A—C9A—N2A	−54.1 (9)	N1B—C8B—C9B—N2B	−53.1 (9)
C12A—N2A—C10A—C11A	139.4 (7)	C12B—N2B—C10B—C11B	132.7 (7)
C9A—N2A—C10A—C11A	−52.0 (9)	C9B—N2B—C10B—C11B	−55.6 (9)
C5A—N1A—C11A—C10A	168.5 (6)	C5B—N1B—C11B—C10B	165.3 (6)
C8A—N1A—C11A—C10A	−55.3 (8)	C8B—N1B—C11B—C10B	−58.8 (8)
N2A—C10A—C11A—N1A	52.7 (9)	N2B—C10B—C11B—N1B	57.2 (9)
C9A—N2A—C12A—O3A	3.5 (11)	C10B—N2B—C12B—O3B	−3.5 (11)
C10A—N2A—C12A—O3A	171.6 (7)	C9B—N2B—C12B—O3B	−174.1 (7)
C9A—N2A—C12A—O4A	−178.7 (6)	C10B—N2B—C12B—O4B	178.4 (6)
C10A—N2A—C12A—O4A	−10.5 (10)	C9B—N2B—C12B—O4B	7.7 (10)
C13A—O4A—C12A—O3A	1.5 (9)	C13B—O4B—C12B—O3B	−0.8 (10)
C13A—O4A—C12A—N2A	−176.4 (6)	C13B—O4B—C12B—N2B	177.4 (6)
C12A—O4A—C13A—C14A	176.7 (6)	C12B—O4B—C13B—C14B	−178.3 (6)

### Hydrogen-bond geometry (Å, º)

**Table:**

*D*—H···*A*	*D*—H	H···*A*	*D*···*A*	*D*—H···*A*
O1*A*—H1*A*···O2*B*	0.84	2.14	2.973 (8)	170
C8*A*—H8*AB*···O3*B*^i^	0.99	2.56	3.225 (11)	124
O1*B*—H1*B*···O2*A*	0.84	2.11	2.934 (8)	168

Symmetry code: (i) −*x*+1, −*y*+1, *z*+1/2.
